# Supplementary figures and images for: Food insecurity increases energetic efficiency, not food consumption: an exploratory study in European starlings
Source: PeerJ. 2021 May 28;9:e11541. doi: 10.7717/peerj.11541 (PMC8166238; doi:10.7717/peerj.11541)

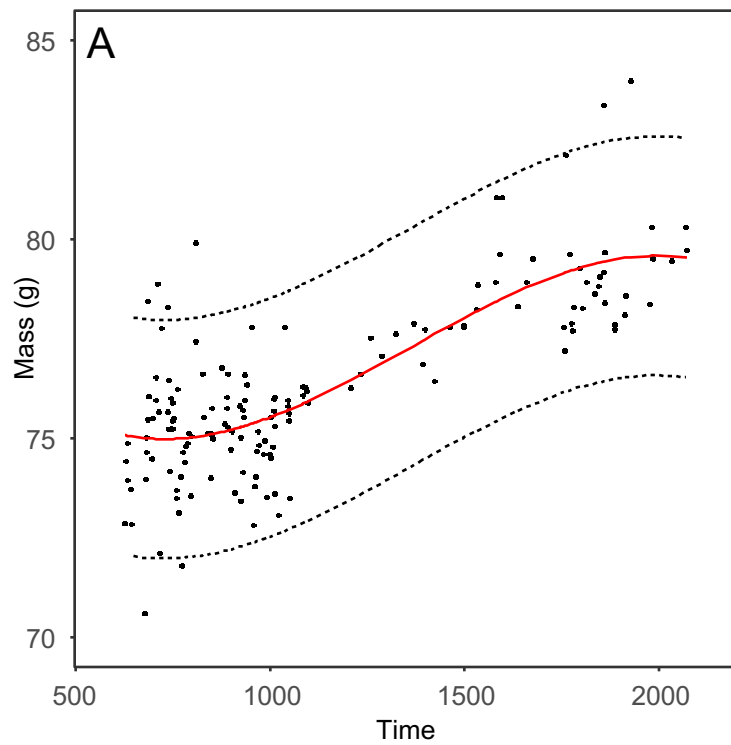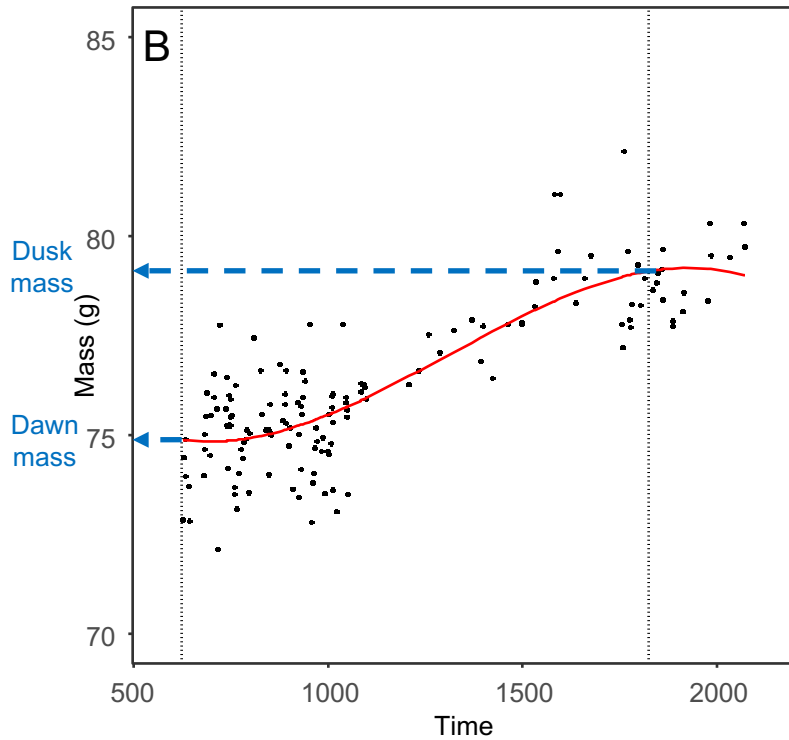

Supplement: Supplemental Information 2 — (A) Scatterplot of body mass against time of day for a single bird on a single day in experiment 4. The red line shows the polynomial fit to the raw data. The dashed black lines show the 3 g band either side of the fitted line; masses lying outside this band were deleted. (B) Scatter plot of the cleaned body mass data. The red line shows the slightly modified polynomial fit after removing the outliers. The vertical dotted lines show the times designated as dawn and dusk in experiment 4 (0615 and 1815 respectively) and the blue arrows show the estimates of dawn and dusk mass for this bird on this day. [file peerj-09-11541-s002.pdf]

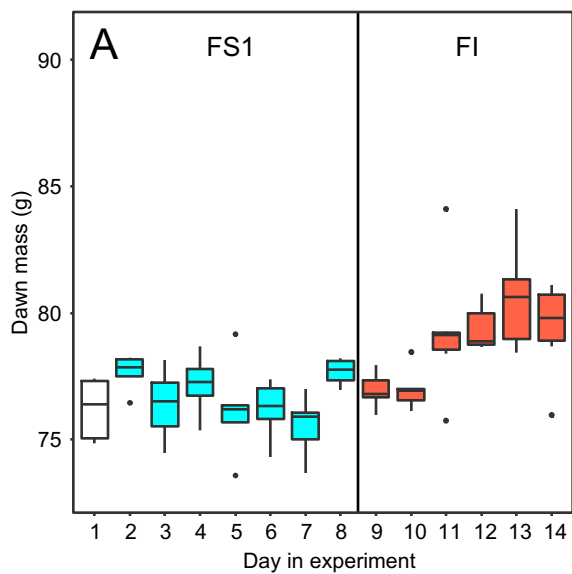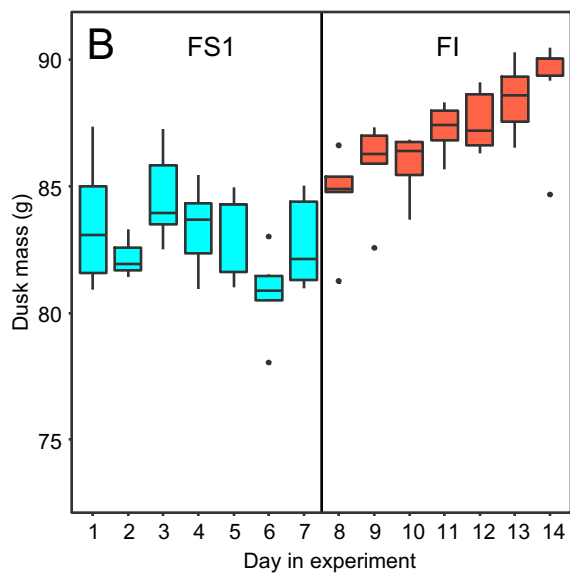

Supplement: Supplemental Information 3 — (A) Dawn mass (g) and (B) dusk mass (g). Graphs are box plots of the mass data from experiment 2, with each box corresponding to a single day of data. For dawn mass the treatment is that in place the previous day, whereas for dusk mass the treatment is that in place the same day (the first box of panel A is white because there was no treatment in place the previous day). The vertical lines indicate when treatment changed from FS1 to FI. For display purposes, data were within-subject centred and plotted relative to the grand mean. The n was 6 birds. [file peerj-09-11541-s003.pdf]
