# Supplementary material for: Food insecurity increases energetic efficiency, not food consumption: an exploratory study in European starlings
Source: PeerJ. 2021 May 28;9:e11541. doi: 10.7717/peerj.11541 (PMC8166238; doi:10.7717/peerj.11541)
Supplement: Supplemental Information 4 [file peerj-09-11541-s004.docx]

**Table S1**: Details of the four experiments.

| Exp. | No. birds | Sex | Age | Lights on | Light: Dark hours | Birds/aviary | SFS/aviary | Duration of FI (weeks) | Reinstatement of FS? |
| --- | --- | --- | --- | --- | --- | --- | --- | --- | --- |
| 1 | 6 | M | ~6 years | 0900-1800 | 9:15 | 2 | 1 | 2 | Yes (1 week) |
| 2 | 6 | M | ~6 years | 0900-1800 | 9:15 | 6 | 2 | 1 | no |
| 3 | 6 | M | ~6 years | 0900-1800 | 9:15 | 6 | 2 | 1 | Yes (2 weeks) |
| 4 | 6 | F | ~4 years | 0600-2100 | 15:9 | 2 | 1 | 1 FI_low_ and 1 FI_high_ | no |

Notes:

- The six birds used in experiments 1-3 were in reproductive condition at the time of the experiments.
- The birds used in experiment 4 had been permanently maintained on long days since hand-rearing and were thus non-reproductive and non-photosensitive.
