# Supplementary material for: Food insecurity increases energetic efficiency, not food consumption: an exploratory study in European starlings
Source: PeerJ. 2021 May 28;9:e11541. doi: 10.7717/peerj.11541 (PMC8166238; doi:10.7717/peerj.11541)
Supplement: Supplemental Information 6 [file peerj-09-11541-s006.docx]

**Table S3.** Summary of linear mixed models of effects of food insecurity on dusk^1^ mass (g).

| Expt. | Random effects | Treatment effect^2^ | Parameter estimate^3^ | 95% CI | Test statistic and df | value | p-value |
| --- | --- | --- | --- | --- | --- | --- | --- |
| 1 | Aviary/bird | Overall^4^ |  |  | F2,138 | 2.86 | 0.061 |
|  |  | FI v. FS1 | βFI = 0.82 | -0.29 to 1.93 | t138 | 1.45 | 0.150 |
|  |  | FS2 v. FS1 | βFS2 = -0.50 | -1.76 to 0.75 | t138 | -0.79 | 0.432 |
|  |  | FI v. FS2 | βFI = 1.32 | 0.20 to 2.45 | t138 | 2.30 | 0.023* |
| 2 | Bird | Overall | βFI = 4.25 | 3.33 to 5.18 | F1,76 | 82.01 | <0.001*** |
| 3 |  | Overall |  |  | F2,151 | 23.90 | <0.001*** |
|  |  | FI v. FS1 | βFI = 2.93 | 2.04 to 3.81 | t151 | 6.50 | <0.001*** |
|  |  | FS2 v. FS1 | βFS2 = 0.72 | -0.05 to 1.49 | t151 | 1.83 | 0.069 |
|  |  | FI v. FS2 | βFI = 2.21 | 1.44 to 2.97 | t151 | 5.64 | <0.001*** |
| 4 |  | Overall |  |  | F2,94 | 4.01 | 0.021* |
|  |  | FI_low_ v. FS1 | βFI_low_ = 0.79 | -0.12 to 1.71 | t94 | 1.69 | 0.094 |
|  |  | FS_high_ v. FS1 | βFI_high_ = 1.33 | 0.41 to 2.27 | t94 | 2.82 | 0.006** |
|  |  | FI_high_ v. FI_low_ | βFI_high_ = 0.55 | -0.36 to 1.45 | t94 | 1.19 | 0.239 |

Notes:

1. Mass estimated at 1800 hours for expt. 1-3. and 1815 for expt. 4. These times equate to 9 hours after dawn and 12 hours after dawn respectively. Unit of analysis is bird day.
2. The reference category is always given second.
3. For comparisons involving food insecurity, the parameter estimates are always expressed such that a positive number means that birds gained weight under greater FI.
4. Overall tests: type III ANOVA with Satterthwaite’s method.
5. * p < 0.05, ** p < 0.01, *** p < 0.001.
