# Supplementary material for: Food insecurity increases energetic efficiency, not food consumption: an exploratory study in European starlings
Source: PeerJ. 2021 May 28;9:e11541. doi: 10.7717/peerj.11541 (PMC8166238; doi:10.7717/peerj.11541)
Supplement: Supplemental Information 7 [file peerj-09-11541-s007.docx]

**Table S4.** Summary of meta-analysis^1^ of body mass, food consumption and energetic efficiency in experiments 1-4.

| Dependent variable | Test of random-effects model | | | | Heterogeneity statistics^2,3^ | | | | |
| --- | --- | --- | --- | --- | --- | --- | --- | --- | --- |
|  | Estimate | 95% CI | z | p-value | ρ | τ^2^ | Test for heterogeneity | | |
|  |  |  |  |  |  |  | Q | df | p-value |
| Dawn mass (g) | 0.52 | -0.12 to 1.17 | 1.59 | 0.113 | -0.35 | 0.95 | 29.79 | 7 | <0.001*** |
| Dusk mass (g) | 1.51 | 0.78 to 2.25 | 4.05 | <0.001*** | -0.47 | 3.14 | 67.07 | 7 | <0.001*** |
| Daily food consumption (g) | -2.50 | -3.13 to -1.87 | -7.77 | <0.001*** | -0.49 | 3.20 | 61.76 | 7 | <0.001*** |
| Energetic efficiency | 0.85 | 0.65 to 1.06 | 8.17 | <0.001*** | -0.53 | 0.20 | 29.35 | 7 | <0.001*** |

Notes:

1. Unit of analysis is aviary. As the dataset includes multiple effects from the same experiment for experiments 1 and 4, we used multilevel models containing nested random effects of association and experiment.
2. τ^2^ estimates the heterogeneity between associations.
3. ρ estimates the intra-class correlation coefficient between the associations from the same experiment.
4. *** p < 0.001.
