# Supplementary material for: Food insecurity increases energetic efficiency, not food consumption: an exploratory study in European starlings
Source: PeerJ. 2021 May 28;9:e11541. doi: 10.7717/peerj.11541 (PMC8166238; doi:10.7717/peerj.11541)
Supplement: Supplemental Information 8 [file peerj-09-11541-s008.docx]

**Table S5.** Summary of linear mixed models of effects of food insecurity on foraging variables in experiment 4.

| Dependent variable | Treatment effect^3^ | Parameter estimate | 95% CI | Test statistic | | p-value |
| --- | --- | --- | --- | --- | --- | --- |
|  |  |  |  | Type, df | Value |  |
| Peck rate^1^ (pecks.hr^-1^) | Overall^4^ |  |  | F2,118 | 87.16 | <0.001*** |
|  | FI_low_ v. FS1 | βFI_low_ = 8.81 | 5.01 to 12.62 | t118 | 4.54 | <0.001*** |
|  | FS_high_ v. FS1 | βFI_high_ = 25.27 | 21.46 to 29.07 | t118 | 13.01 | <0.001*** |
|  | FI_high_ v. FI_low_ | βFI_high_ = 16.46 | 12.65 to 20.26 | t118 | 8.47 | <0.001*** |
| Reinforcement rate^1^ (reinforcements.hr^-1^) | Overall |  |  | F2,118 | 32.95 | <0.001*** |
|  | FI_low_ v. FS1 | βFI_low_ = -5.51 | -7.32 to -3.71 | t118 | -5.97 | <0.001*** |
|  | FS_high_ v. FS1 | βFI_high_ = -7.16 | -8.97 to -5.35 | t118 | -7.75 | <0.001*** |
|  | FI_high_ v. FI_low_ | βFI_high_ = -1.65 | -3.46 to 0.16 | t118 | -1.79 | 0.077 |
| Consumption rate^2^ (g.reinforcement^-1^) | Overall |  |  | F2,55 | 23.07 | <0.001*** |
|  | FI_low_ v. FS1 | βFI_low_ = 0.032 | 0.019 to 0.045 | t55 | 4.89 | <0.001*** |
|  | FS_high_ v. FS1 | βFI_high_ = 0.043 | 0.030 to 0.056 | t55 | 6.57 | <0.001*** |
|  | FI_high_ v. FI_low_ | βFI_high_ = 0.011 | -0.001 to 0.024 | t55 | 1.77 | 0.082 |

Notes:

1. Models include random effects of aviary/bird. Unit of analysis is bird day.
2. Model includes random effect of aviary. Unit of analysis is aviary day.
3. The reference category is always given second.
4. Overall tests: type III ANOVA with Satterthwaite’s method.
5. *** p < 0.001.
