# Supplementary material for: Food insecurity increases energetic efficiency, not food consumption: an exploratory study in European starlings
Source: PeerJ. 2021 May 28;9:e11541. doi: 10.7717/peerj.11541 (PMC8166238; doi:10.7717/peerj.11541)
Supplement: Supplemental Information 9 [file peerj-09-11541-s009.docx]

**Table S6.** Summary of statistical analyses of effects of food insecurity on total food consumption (g/bird/day)^1^.

| Expt. | Random effect(s) | Treatment effect^2^ | Parameter estimate^3^ | 95% CI | Test statistic and df | value | p-value |
| --- | --- | --- | --- | --- | --- | --- | --- |
| 1 | Aviary | Overall^4^ |  |  | F2,79 | 21.73 | <0.001*** |
|  |  | FI v. FS1 | βFI = -2.81 | -3.66 to -1.96 | t79 | -6.47 | <0.001*** |
|  |  | FS2 v. FS1 | βFS2 = -2.39 | -3.37 to -1.41 | t79 | -4.78 | <0.001*** |
|  |  | FI v. FS2 | βFI = -0.41 | -1.26 to 0.44 | t79 | -0.96 | 0.343 |
| 2 | None | Overall | βFI = 0.10 | -1.31- to 1.50 | F1,12 | 0.02 | 0.885 |
| 3 | None | Overall |  |  | F2,25 | 26.62 | <0.001*** |
|  |  | FI v. FS1 | βFI = -2.63 | -3.39 to -1.87 | t25 | -7.12 | <0.001*** |
|  |  | FS2 v. FS1 | βFS2 = -0.89 | -1.55 to -0.23 | t25 | -2.79 | 0.010* |
|  |  | FI v. FS2 | βFI = -1.74 | -2.40 to -1.08 | t25 | -5.43 | <0.001*** |
| 4 | Aviary | Overall |  |  | F2,54 | 8.77 | 0.001*** |
|  |  | FI_low_ v. FS1 | βFI_low_ = -0.71 | -1.88 to 0.46 | t54 | -1.19 | 0.239 |
|  |  | FS_high_ v. FS1 | βFI_high_ = -2.44 | -3.62 to -1.26 | t54 | -4.05 | 0.001*** |
|  |  | FI_high_ v. FI_low_ | βFI_high_ = -1.73 | -2.88 to -0.58 | t54 | -2.94 | 0.005** |

Notes:

1. Unit of analysis is aviary day.
2. The reference category is always given second.
3. For comparisons involving food insecurity the parameter estimates are always expressed such that a negative number means that birds ate less under greater FI.
4. Overall tests: type III ANOVA with Satterthwaite’s method.
5. * p < 0.05, ** p < 0.01, *** p < 0.001.
