# Supplementary material for: Food insecurity increases energetic efficiency, not food consumption: an exploratory study in European starlings
Source: PeerJ. 2021 May 28;9:e11541. doi: 10.7717/peerj.11541 (PMC8166238; doi:10.7717/peerj.11541)
Supplement: Supplemental Information 10 [file peerj-09-11541-s010.docx]

**Table S7.** Summary of linear mixed models of effects of food insecurity on energetic efficiency^1^.

| Expt. | Random effect(s) | Treatment effect^2^ | Parameter estimate^3^ | 95% CI | Test statistic and df | value | p-value |
| --- | --- | --- | --- | --- | --- | --- | --- |
| 1 | Aviary | Overall^4^ |  |  | F2,74 | 17.5 | <0.001*** |
|  |  | FI v. FS1 | βFI = 0.93 | 0.61 to 1.25 | t74 | 5.72 | <0.001*** |
|  |  | FS1 v. FS2 | βFSI = 0.86 | 0.48 to 1.24 | t73 | 4.42 | <0.001*** |
|  |  | FI v. FS2 | βFI = 0.07 | -0.26 to 0.41 | t74 | 0.43 | 0.668 |
| 2 | None | Overall | βFI = 0.16 | -0.36 to 0.67 | F1,11 | 0.45 | 0.515 |
| 3 | None | Overall |  |  | F2,24 | 27.70 | <0.001*** |
|  |  | FS1 v. FI | βFI = 0.97 | 0.69 to 1.26 | t24 | 7.13 | <0.001*** |
|  |  | FS1 v. FS2 | βFS2 = 0.28 | 0.03 to 0.53 | t24 | 2.32 | 0.029* |
|  |  | FI v. FS2 | βFI = 0.70 | 0.45 to 0.94 | t24 | 5.81 | <0.001*** |
| 4 | Aviary | Overall |  |  | F2,55 | 7.99 | <0.001*** |
|  |  | FI_low_ v. FS1 | βFI_low_ = 0.38 | -0.14 to 0.91 | t55 | 1.43 | 0.160 |
|  |  | FS_high_ v. FS1 | βFI_high_ = 1.14 | 0.58 to 1.72 | t55 | 3.95 | 0.001*** |
|  |  | FI_high_ v. FI_low_ | βFI_high_ = 0.76 | 0.21 to 1.31 | t55 | 2.71 | 0.009** |

Notes:

1. Defined as the ratio of the mean dawn mass of the starlings in a room on a given day to the mean mass of food consumed per bird in the room in the previous 24 hours. The higher this ratio, the greater the body mass maintained per gram of food consumed. Unit of analysis is aviary day.
2. The reference category is always given second.
3. For comparisons involving food insecurity the parameter estimates are always expressed such that a positive number means greater efficiency under greater FI.
4. Overall tests: type III ANOVA with Satterthwaite’s method.
5. * p < 0.05, ** p < 0.01, *** p < 0.001.
