# Supplementary material for: Food insecurity increases energetic efficiency, not food consumption: an exploratory study in European starlings
Source: PeerJ. 2021 May 28;9:e11541. doi: 10.7717/peerj.11541 (PMC8166238; doi:10.7717/peerj.11541)
Supplement: Supplemental Information 11 [file peerj-09-11541-s011.docx]

**Table S8.** Summary of linear mixed models of effects of food insecurity on energy density of guano (MJ/kg).

| Expt. | Random effect(s) | Treatment effect^1^ | Parameter estimate | 95% CI | Test statistic and df | value | p-value |
| --- | --- | --- | --- | --- | --- | --- | --- |
| 1 | Aviary/sample | Overall |  |  | F_2,19_ | 3.84 | 0.051 |
|  |  | FI v. FS1 | βFI = -0.17 | -0.33 to  -0.01 | T_19_ | -2.12 | 0.047* |
|  |  | FS2 v. FS1 | βFS2 =  -0.23 | -0.41 to  -0.05 | T_19_ | -2.51 | 0.022* |
|  |  | FI v. FS2 | βFI = 0.06 | -0.10 to 0.22 | T_19_ | 0.77 | 0.452 |
| 3 | Sample | Overall |  |  | F_2,53_ | 6.82 | 0.002** |
|  |  | FI v. FS1 | βFI = -0.07 | -0.22 to 0.09 | t_53_ | -0.84 | 0.403 |
|  |  | FS2 v. FS1 | βFS2 = -0.24 | -0.37 to  -0.10 | t_53_ | -3.42 | 0.001** |
|  |  | FI v. FS2 | βFI = 0.17 | 0.03 to 0.31 | t_53_ | 2.45 | 0.018* |

Notes:

1. The unit of analysis is 3-day pooled samples from each aviary in experiment 1 (n = 48) and pooled daily samples from the single aviary in experiment 3 (n = 56). There were two technical replicates for each sample and sample is thus included as a random effect in both models.
2. For the pairwise comparisons, the reference category is always given second.
3. For comparisons involving FI the parameter estimates are always expressed such that a negative number means the guano contained less energy under FI.
4. Overall tests: type III ANOVA with Satterthwaite’s method.
5. * p < 0.05, ** p < 0.01.
