# Supplementary material for: Food insecurity increases energetic efficiency, not food consumption: an exploratory study in European starlings
Source: PeerJ. 2021 May 28;9:e11541. doi: 10.7717/peerj.11541 (PMC8166238; doi:10.7717/peerj.11541)
Supplement: Supplemental Information 12 [file peerj-09-11541-s012.docx]

**Table S9.** Summary of linear mixed models of effects of food insecurity on the proportion of scans in which a bird was roosting^1^.

| Expt. | Random effect(s) | Treatment effect^2^ | Parameter estimate^3^ | 95% CI | Test statistic and df | value | p-value |
| --- | --- | --- | --- | --- | --- | --- | --- |
| 1 | Aviary/day | Overall^4^ |  |  | F2,76 | 11.98 | <0.001*** |
|  |  | FI v. FS1 | βFI = -0.06 | -0.16 to 0.047 | t76 | -1.06 | 0.293 |
|  |  | FS2 vs FS1 | βFS2 = 0.21 | 0.09 to 0.34 | t76 | 3.39 | 0.001** |
|  |  | FI v. FS2 | βFI = -0.27 | -0.38 to -0.16 | t76 | -4.87 | <0.001*** |
| 2 | Day | Overall | βFI = 0.08 | -0.02 to 0.18 | F1,24 | 2.50 | 0.127 |
| 3 | Day | Overall |  |  | F2,23 | 6.49 | 0.006** |
|  |  | FI vs FS1 | βFI = 0.17 | 0.08 to 0.27 | t24 | 3.47 | 0.002** |
|  |  | FS2 vs FS1 | βFS2 = 0.12 | 0.04 to 0.20 | t23 | 2.79 | 0.011* |
|  |  | FI vs FS2 | βFI = 0.05 | -0.03 to 0.13 | t23 | 1.19 | 0.245 |

Notes:

1. The unit of analysis is aviary day.
2. The dependent variable (proportion of scans in which birds were roosting) was arcsine square root transformed prior to analysis.
3. Given that behaviour was measured between 0900-1100, and thus before any treatment effect has been experienced on the current day, the predictor variable used in the models is the treatment to which the birds were exposed on the previous day. The reference category is always given second.
4. For comparisons involving food insecurity the parameter estimates are expressed such that a positive number means that birds roost more under FI.
5. Overall tests: type III ANOVA with Satterthwaite’s method.
6. * p < 0.05, ** p < 0.01, *** p < 0.001.
